# Supplementary material for: Undernutrition and Feeding Difficulties Among Children with Disabilities in Uganda: A Cross-Sectional Study
Source: Nutrients. 2026 Jan 8;18(2):200. doi: 10.3390/nu18020200 (PMC12844944; doi:10.3390/nu18020200)
Supplement: Supplementary file 1 [file nutrients-18-00200-s001.zip › Nutrients_Supplementary Materials_Feeding Questions.pdf]

# Supplementary Materials – Feeding Questions

## Assessment of Feeding Practices

*(Not all questions are administered for every child. The assessor is guided through a tailored set of questions based on the child's age and the tool used to feed the child.)*

1. How is the child fed? [Bottle, Breast, Spoon/Fork, Cup, Fingers]

*Selection: Spoon, cup, fingers*

2. When feeding the child, how often do you wait for them to show they are ready before offering the next bite? [Always, Sometimes, Never, Does not apply]
3. When using a spoon to feed the child, how often is it a small one? [Always, Sometimes, Never, Does not apply]
4. How often do you talk to/sing to the child during meal time? [Always, Sometimes, Never, Does not apply]
5. When feeding the child, how often do you sit so you are eye-level with the child? [Always, Sometimes, Never, Does not apply]
6. How often does the child have mealtime with others? [Always, Sometimes, Never, Does not apply]

*Selection: Breast*

7. How often do you hold infant with head supported while breastfeeding? [Always, Sometimes, Never, Does not apply]
8. How often do you talk to or sing to infant when feeding? [Always, Sometimes, Never, Does not apply]
9. How often do you follow infants' cues to stop or pause feeding? [Always, Sometimes, Never, Does not apply]

*Selection: Bottle*

10. When bottle feeding, how often do you use bottle nipples that are intact? [Always, Sometimes, Never, Does not apply]
11. While bottle feeding, how often do you offer infants breaks to burp? [Always, Sometimes, Never, Does not apply]
12. How often do you hold infant with head supported while bottle feeding? [Always, Sometimes, Never, Does not apply]
13. How often do you talk to or sing to infant when feeding? [Always, Sometimes, Never, Does not apply]
14. How often do you follow infants' cues to stop or pause feeding? [Always, Sometimes, Never, Does not apply]

## Screening for Feeding Difficulties

*(Not all questions are administered for every child. The assessor is guided through a tailored set of questions based on the child's age, health condition, and responses to previous feeding questions.)*

1. Does this child have any difficulties feeding? [Yes, No, I don't Know]
2. Who typically feeds the child? [Caregiver, Child]
3. How much help does the child usually need to get food or liquid to their mouth? [Full support needed, Some support needed, No support needed]
4. How is the child fed? [Bottle, Breast, Spoon/Fork, Cup, Fingers]
5. What is typically offered at mealtimes? [Formula and/or breastmilk, Thin liquids (not formula), Puree, Mashed, Soft & bite-sized, Regular foods]
6. Has child been offered an opportunity to hold spoon or touch food with hands? [Yes, No]
7. Has child been offered a spoon? [Yes, No]
8. Has child been offered a cup? [Yes, No]
9. Has child been unsuccessful drinking from a bottle? [Yes, No]
10. Has child been offered purees? [Yes, No]
11. Has child been offered fork-mashed foods? [Yes, No]
12. Is child chewing food offered? [Yes, No]
13. How many bottles does child usually drink per day? [2-12]
14. How much prepared formula is typically in each bottle? [60 ml, 90 ml, 120 ml, 150 ml, 180 ml, 210 ml, 240 ml]
15. How much of each bottle does child typically finish? [All, ¾, ½, ¼, Less than ¼]
16. How many calories per milliliter does the formula provide? [67 kcal/100 ml, 73 kcal/100 ml, 80 kcal/100 ml, 100 kcal/100 ml, I do not know]
17. How long does mealtime typically last? [Less than 10 minutes, 10-30 minutes, More than 30 minutes]
18. Does the child frequently cough or choke? [Yes, No]
    - If yes, when does this happen? [With liquids from a bottle, With liquids from a cup, With solids from a spoon, With all foods and liquids, After mealtime is finished, Throughout the day]
