# Supplementary material for: Undernutrition and Feeding Difficulties Among Children with Disabilities in Uganda: A Cross-Sectional Study
Source: Nutrients. 2026 Jan 8;18(2):200. doi: 10.3390/nu18020200 (PMC12844944; doi:10.3390/nu18020200)
Supplement: Supplementary file 1 [file nutrients-18-00200-s001.zip › Nutrients_Supplementary Materials_TableS1.pdf]

Supplementary Materials

Table S1. Demographics of children with outliers in anthropometric z-scores

Outliers in weight for age z-scores (WAZ): < -6 or > +5 (n=6)

WAZ	Age (months)	Sex	Health Condition
-7	4.862423	Male	Cleft lip/palate
-6.18	15.86858	Male	Cerebral palsy
-6.14	13.33881	Female	Other DD
-6.12	40.93634	Male	Cerebral palsy
-6.11	25.72485	Female	Cleft lip/palate
-6.02	1.971253	Female	Cleft lip/palate
9.71	6.505134	Male	Cerebral palsy

Outliers in length/height for age z-scores (L/HAZ): < -6 or > +6 (n=13)

L/HAZ	Age (months)	Sex	Health Condition
-7.61	1.971253	Female	Cleft lip/palate
-6.99	40.93634	Male	Cerebral palsy
-6.95	4.862423	Male	Cleft lip/palate
-6.91	25.72485	Female	Cleft lip/palate
-6.82	15.14579	Male	Cleft lip/palate
-6.7	15.86858	Male	Cerebral palsy
-6.45	11.53183	Male	Cleft lip/palate
-6.31	6.439425	Female	Cleft lip/palate
6.06	4.435318	Male	Cleft lip/palate
6.99	12.61602	Female	Cerebral palsy
8.44	12.74743	Male	Cleft lip/palate
9.23	22.6037	Male	Cerebral palsy
19.57	12.81314	Male	Cerebral palsy

Outliers in weight for length/height z-scores (WL/HZ): < -5 or > +5 (n=6)

WFL/H	Age (months)	Sex	Health Condition
-7.21	47.50719	Female	Cerebral palsy
-6.91	34.33265	Male	Cerebral palsy
-6.87	15.80287	Male	Cleft lip/palate
-6.12	12.61602	Female	Cerebral palsy
-5.67	12.74743	Male	Cleft lip/palate
-5.26	.8213552	Male	Cleft lip/palate
