# Supplementary material for: Undernutrition and Feeding Difficulties Among Children with Disabilities in Uganda: A Cross-Sectional Study
Source: Nutrients. 2026 Jan 8;18(2):200. doi: 10.3390/nu18020200 (PMC12844944; doi:10.3390/nu18020200)
Supplement: Supplementary file 1 [file nutrients-18-00200-s001.zip › Nutrients_Supplementary Materials_TableS2.pdf]

Supplementary Materials

Table S2. Prevalence of undernutrition among children birth to 10 years old with cerebral palsy (n=165)

	Underweight[1]		Stunting[1]		Wasting[1,2]				Anemia[3]	
	**WAZ (0-120 months) N=158**		**L/HAZ (0-120 months) N=110**		**WL/HZ (0-59 months) N=94**		**MUAC in cm (6-120 months) N=150**		**Hemoglobin (g/dl) (6-120 months) N=90**	
**Variables**	**N (%)**	***p*-Value**	**N (%)**	***p*-Value**	**N (%)**	***p*-Value**	**N (%)**	***p*-Value**	**N (%)**	***p*-Value**
**Undernutrition**		--		--		--		--		--
No	75 (47.5)		65 (59.1)		58 (61.7)		121 (80.7)		61 (67.8)	
Mild/Moderate	33 (20.9)		19 (17.3)		18 (19.2)		20 (13.3)		29 (32.2)	
Severe	50 (31.7)		26 (23.6)		18 (19.2)		9 (6.0)		0 (0.0)	
**Sex**		0.440		0.406		0.899		0.308		0.218
Female	36/64 (56.3)		23/51 (45.1)		16/41 (39.0)		9/59 (15.3)		8/33 (24.2)	
Male	47/94 (50.0)		22/59 (37.3)		20/53 (37.7)		20/91 (22.0)		21/57 (36.8)	
**Age**		0.308		0.722		0.191		0.989		0.157
<6 months	3/7 (42.9)		2/7 (28.6)		2/7 (28.6)		--		--	
6-11 months	8/23 (34.8)		8/23 (34.8)		5/23 (21.7)		5/24 (20.8)		6/13 (46.2)	
12-23 months	18/36 (50.0)		13/27 (48.2)		14/28 (50.0)		7/37 (18.9)		7/21 (33.3)	
24–59 months	33/56 (57.1)		14/37 (37.8)		15/36 (41.7)		10/55 (18.2)		13/34 (38.2)	
60–120 months	22/36 (61.1)		8/16 (50.0)		--		7/34 (20.6)		3/22 (13.6)	
**Number of health conditions**		0.774		0.156		**0.036**		0.607		0.910
Cerebral palsy only	66/127 (52.0)		34/90 (37.8)		33/76 (43.4)		22/119 (18.5)		23/72 (31.9)	
Cerebral palsy + other conditions	17/31 (54.8)		11/21 (55.0)		3/18 (16.7)		7/31 (22.6)		6/18 (33.3)	
**Reported coughing & choking**		0.692		0.275		0.272		0.752		0.873
No	61/114 (53.5)		36/82 (43.9)		28/67 (41.8)		21/105 (20.0)		22/69 (31.9)	
Yes	22/44 (50.0)		9/28 (32.1)		8/287 (29.6)		8/45 (17.8)		6/20 (30.0)	
**Reported infection**		0.646		0.964		0.684		**0.017**		--
No	50/93 (53.8)		28/69 (40.6)		24/59 (40.7)		11.87 (12.6)		--[4]	
Yes	31/62 (50.0)		16/39 (41.0)		12/33 (36.4)		17/60 (28.3)		--	
**Functional difficulties**		0.400		0.208		0.324		0.139		0.096
No	4/9 (44.4)		1/6 (16.7)		1/5 (20.0)		0/9 (0.0)		0/6 (0.0)	
Yes	49/83 (59.0)		20/46 (43.5)		13/30 (43.3)		16/80 (20.0)		16/49 (32.7)	
**Functional difficulty in mobility**		0.073		**0.020**		0.071		**0.032**		0.052
No	7/18 (38.9)		2/14 (14.3)		1/8 (12.5)		0/17 (0.0)		1/13 (7.7)	
Yes	46/74 (62.2)		19/38 (50.0)		13/27 (48.2)		16/72 (22.2)		15/42 (35.7)	

L/HAZ: Length/Height-for-age *z*-score; MUAC: Mid-upper arm circumference; WAZ: Weight-for-age *z*-score; WL/HZ: Weight-for-length/height *z*-score. Statistical analyses using Pearson's chi-squared. *P*-values shown in bold are statistically significant (< 0.05).

[1]Classification of underweight, stunting, and wasting (WL/HZ) was based on *z*-score thresholds: normal (≥ -2), moderate (< -2 and ≥ -3), and severe (< -3).

[2]Classification of wasting was also based on MUAC cut-offs: for ages 6-59 months: normal (≥ 12.5 cm), moderate (< 12.5 cm and ≥ 11.5 cm), and severe (< 11.5 cm); and for ages 60-120 months: normal (≥ 14.5 cm), moderate (< 14.5 and ≥ 13.5 cm), and severe (< 13.5 cm).

[3]Classification of anemia was based on age-specific hemoglobin cut-offs: for 6-23 months: mild (9.5–10.4 g/dL), moderate (7.0–9.49 g/dL), and severe (<7.0 g/dL); for 24-59 months: mild (10.0–10.9 g/dL), moderate (7.0–9.9 g/dL), and severe (<7.0 g/dL); and for 5–11 years: mild (11.0–11.4 g/dL), moderate (8.0–10.9 g/dL), and severe (<8.0 g/dL).

[4]Hemoglobin was not measured in children with reported infections.
