# Supplementary material for: Undernutrition and Feeding Difficulties Among Children with Disabilities in Uganda: A Cross-Sectional Study
Source: Nutrients. 2026 Jan 8;18(2):200. doi: 10.3390/nu18020200 (PMC12844944; doi:10.3390/nu18020200)
Supplement: Supplementary file 1 [file nutrients-18-00200-s001.zip › Nutrients_Supplementary Materials_TableS3.pdf]

Supplementary Materials

Table S3. Prevalence of undernutrition among children birth to 10 years old with cleft lip and/or palate (n=237)

	**Underweight**[1]		**Stunting**[1]		**Wasting**[1,2]		**Anemia**[3]		**Underweight**[1]	
	**WAZ (0-120 months) N=231**		**L/HAZ (0-120 months) N=220**		**WL/HZ (0-59 months) N=218**		**MUAC in cm (6-120 months) N=103**		**WAZ (0-120 months) N=68**	
**Variables**	**N (%)**	***p*-Value**	**N (%)**	***p*-Value**	**N (%)**	***p*-Value**	**N (%)**	***p*-Value**	**N (%)**	***p*-Value**
**Undernutrition**		--		--		--		--		--
No	137 (59.3)		135 (61.4)		176 (80.7)		93 (90.3)		35 (51.5)	
Mild/Moderate	40 (17.3)		42 (19.1)		26 (11.9)		6 (5.8)		33 (48.5)	
Severe	54 (23.4)		43 (19.6)		16 (7.3)		4 (3.9)		0 (0.0)	
**Sex**		0.055		0.292		**0.010**		0.923		0.230
Female	36/106 (34.0)		36/103 (35.0)		12/101 (11.9)		5/53 (9.4)		15/36 (41.7)	
Male	58/125 (46.4)		49/117 (41.9)		30/117 (25.6)		5/50 (10.0)		18/32 (56.5)	
**Age**		0.364		0.139		0.885		0.290		0.171
<6 months	57/128 (44.5)		45/123 (36.6)		26/124 (21.0)		--		--	
6-11 months	18/55 (32.7)		17/53 (32.1)		10/55 (18.2)		5/55 (9.1)		17/35 (48.6)	
12-23 months	13/29 (44.8)		16/26 (61.5)		4/26 (15.4)		1/28 (3.6)		8/18 (44.4)	
24–59 months	3/13 (23.1)		5/13 (38.5)		2/13 (15.4)		3/14 (21.4)		4/11 (36.4)	
60–120 months	3/6 (50.0)		2/5 (40.0)		--		1/6 (16.7)		4/4 (100.0)	
**Number of health conditions**		0.065		**0.011**		0.061		0.247		0.506
Cleft lip/palate only	81/209 (38.8)		72/200 (36.0)		35/198 (17.7)		8/93 (8.6)		30/60 (50.0)	
Cleft lip/palate + other conditions	13/22 (59.1)		13/20 (65.0)		7/20 (35.0)		2/10 (20.0)		3/8 (37.5)	
**Reported feeding difficulties**[4]		**0.049**		0.057		**0.020**		**0.030**		0.068
No	74/196 (37.8)		69/190 (36.3)		30/189 (15.9)		5/82 (6.1)		23/54 (42.6)	
Yes	17/30 (56.7)		14/25 (56.0)		9/26 (34.6)		4/18 (22.2)		8/11 (72.7)	
**At risk for feeding difficulties**[5]		0.115		**0.014**		0.138		0.208		0.111
No	46/127 (36.2)		39/122 (32.0)		19/123 (15.5)		1/27 (3.7)		6/19 (31.6)	
Yes	47/101 (46.5)		46/95 (48.4)		22/94 (23.4)		9/74 (12.2)		25/47 (53.2)	
**Reported coughing & choking**		**0.017**		0.577		0.101		0.117		0.250
No	69/186 (37.1)		69/180 (38.3)		30/178 (16.9)		5/72 (6.9)		22/51 (43.1)	
Yes	24/42 (57.1)		16/37 (43.2)		11/39 (28.2)		5/29 (17.2)		9/15 (60.0)	
**Reported infection**		**0.015**		0.824		0.227		0.342		--
No	56/160 (35.0)		59/155 (38.1)		25/152 (16.5)		5/65 (7.7)		--[6]	
Yes	36/69 (52.2)		25/63 (39.7)		15/64 (23.4)		5/37 (13.5)		--	
**Functional difficulties**		0.848		0.361		0.207		0.149		0.782
No	4/12 (33.3)		6/12 (50.0)		1/9 (11.1)		1/12 (8.3)		4/8 (50.0)	

Yes	3/8 (37.5)		2/7 (28.6)		2/5 (40.0)		3/9 (33.3)		4/7 (57.1)	
**Exclusive breastfeeding (< 6 months old)**		**0.008**		**0.040**		0.201		--		--
No	48/94 (51.1)		38/90 (42.2)		21/91 (23.1)		--[7]		--[7]	
Yes	8/33 (24.2)		7/32 (21.9)		4/32 (12.5)		--		--	
**Feeding method (< 12 months old)**		**0.008**		**0.016**		0.339		0.428		0.344
Breastfed	11/43 (25.6)		10/42 (23.8)		5/42 (11.9)		0/6 (0.0)		1/4 (25.0)	
Bottle-fed	44/81 (54.3)		37/78 (47.4)		20/79 (25.3)		2/21 (9.5)		8/14 (57.1)	
Breastfed & bottle-fed	10/29 (34.5)		10/29 (34.5)		5/29 (17.2)		1/3 (33.3)		0/2 (0.0)	
Neither (used cup, spoon, fingers)	9/29 (31.0)		5/26 (19.2)		5/28 (17.9)		2/25 (8.0)		8/15 (53.3)	
**Feeding practices among infants < 12 months old**		0.073		0.079		0.591		0.341		0.085
Less than ideal	51/113 (45.1)		42/108 (38.9)		24/110 (21.8)		4/43 (9.3)		15/27 (55.6)	
Ideal	19/61 (31.1)		15/59 (25.4)		11/60 (18.3)		0/9 (0.0)		1/6 (16.7)	

L/HAZ: Length/Height-for-age *z*-score; MUAC: Mid-upper arm circumference; WAZ: Weight-for-age *z*-score; WL/HZ: Weight-for-length/height *z*-score. Statistical analyses using Pearson's chi-squared. *P*-values shown in bold are statistically significant (< 0.05).

[1]Classification of underweight, stunting, and wasting (WL/HZ) was based on *z*-score thresholds: normal (≥ -2), moderate (< -2 and ≥ -3), and severe (< -3).

[2]Classification of wasting was also based on MUAC cut-offs: for ages 6-59 months: normal (≥ 12.5 cm), moderate (< 12.5 cm and ≥ 11.5 cm), and severe (< 11.5 cm); and for ages 60-120 months: normal (≥ 14.5 cm), moderate (< 14.5 and ≥ 13.5 cm), and severe (< 13.5 cm).

[3]Classification of anemia was based on age-specific hemoglobin cut-offs: for 6-23 months: mild (9.5–10.4 g/dL), moderate (7.0–9.49 g/dL), and severe (<7.0 g/dL); for 24-59 months: mild (10.0–10.9 g/dL), moderate (7.0–9.9 g/dL), and severe (<7.0 g/dL); and for 5–11 years: mild (11.0–11.4 g/dL), moderate (8.0–10.9 g/dL), and severe (<8.0 g/dL).

[4]Feeding difficulties reported by caregiver.

[5]Risk for feeding difficulties as determined by the study feeding screening tool.

[6]Hemoglobin was not measured in children with reported infections.

[7]No observations because not reported for infants < 6 months.
