# Supplementary material for: Undernutrition and Feeding Difficulties Among Children with Disabilities in Uganda: A Cross-Sectional Study
Source: Nutrients. 2026 Jan 8;18(2):200. doi: 10.3390/nu18020200 (PMC12844944; doi:10.3390/nu18020200)
Supplement: Supplementary file 1 [file nutrients-18-00200-s001.zip › Nutrients_Supplementary Materials_TableS4.pdf]

Supplementary Materials

Table S4. Logistic regression models with post-estimation and goodness-of-fit tests for the association of risk for feeding difficulties with underweight in children with disabilities birth to 10 years old (n=402)

**Underweight (WAZ)**									
	**Model 1 (unadjusted)**			**Model 2 (demographics)**			**Model 3 (demographics + health)**		
**Variables**	**OR**	**95% CI**	***p*-Value**	**AOR**	**95% CI**	***p*-Value**	**AOR**	**95% CI**	***p*-Value**
**Risk for feeding difficulties**									
No	Ref.			Ref.			Ref.		
Yes	1.84	1.20-2.80	**0.005**	2.34	1.27-4.31	**0.007**	2.28	1.27-4.00	**0.006**
**Sex**									
Female				Ref.			Ref.		
Male				1.27	0.84-1.91	0.249	1.31	0.86-1.98	0.205
**Age**									
< 6 months				Ref.			Ref.		
6-11 months				0.40	0.20-0.80	**0.010**	0.37	0.18-0.75	**0.006**
12-23 months				0.61	0.29-1.26	0.181	0.61	0.29-1.26	0.183
24–59 months				0.73	0.34-1.55	0.412	0.69	0.32-1.49	0.348
60–120 months				1.10	0.45-2.70	0.828	1.05	0.43-2.58	0.914
**Health conditions**									
Other developmental disabilities				Ref.			Ref.		
Cleft lip/palate				0.83	0.32-2.18	0.711	0.86	0.33-2.28	0.769
Cerebral palsy				0.79	0.29-2.14	0.649	0.87	0.32-2.37	0.784
**Reported infection**									
No							Ref.		
Yes							1.53	0.99-2.35	0.053
**Number of health conditions**									
One							Ref.		
Two or more							1.66	0.90-3.05	0.105
**Post-estimation and goodness-of-fit tests**									
Hosmer–Lemeshow test				H-L $\chi^2_{(8)}$=5.35; *p*-value: 0.719			H-L $\chi^2_{(8)}$=4.60; *p*-value: 0.799		
Area under ROC curve				0.638			0.651		
AIC				551.25			549.02		
BIC				587.22			592.98		
Likelihood-ratio test (Model 3 vs. 2)				LR $\chi^2_{(2)}$=6.23; *p*-value: **0.044**					

AIC: Akaike's information criterion; AOR: Adjust odds ratio; BIC: Bayesian information criterion; H-L: Hosmer–Lemeshow; LR: Likelihood-ratio; OR: odds ratio; Ref: reference group; ROC: Receiver Operating Characteristic; SE: Standard error; WAZ: weight-for-age *z*-score
P-values shown in bold are statistically significant (< 0.05).
