# Supplementary material for: Undernutrition and Feeding Difficulties Among Children with Disabilities in Uganda: A Cross-Sectional Study
Source: Nutrients. 2026 Jan 8;18(2):200. doi: 10.3390/nu18020200 (PMC12844944; doi:10.3390/nu18020200)
Supplement: Supplementary file 1 [file nutrients-18-00200-s001.zip › Nutrients_Supplementary Materials_TableS5.pdf]

Supplementary Materials

Table S5. Logistic regression models with post-estimation and goodness-of-fit tests for the association of risk for feeding difficulties with stunting in children with disabilities birth to 10 years old (n=337)

**Stunting (L/HAZ)**									
	**Model 1 (unadjusted)**			**Model 2 (demographics)**			**Model 3 (demographics + health)**		
**Variables**	**OR**	**95% CI**	***p*-Value**	**AOR**	**95% CI**	***p*-Value**	**AOR**	**95% CI**	***p*-Value**
**Risk for feeding difficulties**									
No	Ref.			Ref.			Ref.		
Yes	1.79	1.12-2.86	**0.015**	2.39	1.24-4.59	**0.009**	2.46	1.26-4.79	**0.008**
**Sex**									
Female				Ref.			Ref.		
Male				1.07	0.68-1.68	0.773	1.18	0.73-1.83	0.545
**Age**									
< 6 months				Ref.			Ref.		
6-11 months				0.60	0.29-1.23	0.162	0.56	0.27-1.17	0.122
12-23 months				1.59	0.73-3.44	0.243	1.69	0.77-3.74	0.191
24–59 months				0.91	0.40-2.08	0.820	0.87	0.37-2.03	0.744
60–120 months				1.43	0.47-4.29	0.528	1.58	0.51-4.88	0.423
**Health conditions**									
Other developmental disabilities				Ref.					
Cleft lip/palate				3.77	0.94-15.18	0.061	4.21	1.03-17.16	**0.045**
Cerebral palsy				2.27	0.57-8.93	0.242	2.19	0.55-8.69	0.265
**Reported infection**									
No							Ref.		
Yes							1.05	0.64-1.73	0.832
**Number of health conditions**									
One							Ref.		
Two or more							3.30	1.62-6.73	**0.001**
**Post-estimation and goodness-of-fit tests**									
Hosmer–Lemeshow test				H-L $\chi^2_{(7)}$= 3.94; *p*-value: 0.787			H-L $\chi^2_{(8)}$=3.86; *p*-value: 0.869		
Area under ROC curve				0.623			0.670		
AIC				450.56			443.32		
BIC				484.94			485.34		
Likelihood-ratio test (Model 3 vs. 2)				LR $\chi^2_{(2)}$=11.24; *p*-value: **0.004**					

AIC: Akaike's information criterion; AOR: Adjust odds ratio; BIC: Bayesian information criterion; H-L: Hosmer–Lemeshow; L/HAZ: Length/height-for-age *z*-score; LR: Likelihood-ratio; OR: odds ratio; Ref: reference group; ROC: Receiver Operating Characteristic; SE: Standard error
P-values shown in bold are statistically significant (< 0.05).
