# Supplementary material for: Undernutrition and Feeding Difficulties Among Children with Disabilities in Uganda: A Cross-Sectional Study
Source: Nutrients. 2026 Jan 8;18(2):200. doi: 10.3390/nu18020200 (PMC12844944; doi:10.3390/nu18020200)
Supplement: Supplementary file 1 [file nutrients-18-00200-s001.zip › Nutrients_Supplementary Materials_TableS6.pdf]

Supplementary Materials

Table S6. Logistic regression models with post-estimation and goodness-of-fit tests for the association of risk for feeding difficulties with wasting in children with disabilities under 5 years old (n=319)

Wasting (WL/HZ)									
	Model 1 (unadjusted)			Model 2 (demographics)			Model 3 (demographics + health)		
**Variables**	**OR**	**95% CI**	***p*-Value**	**AOR**	**95% CI**	***p*-Value**	**AOR**	**95% CI**	***p*-Value**
**Risk for feeding difficulties**									
No	Ref.			Ref.			Ref.		
Yes	2.79	1.56-5.00	**0.001**	2.47	1.12-5.43	**0.025**	2.43	1.10-5.35	**0.028**
**Sex**									
Female				Ref.			Ref.		
Male				1.67	0.97-2.87	0.066	1.66	0.96-2.90	0.070
**Age**									
< 6 months				Ref.			Ref.		
6-11 months				0.49	0.21-1.14	0.100	0.47	0.20-1.11	0.085
12-23 months				0.80	0.33-1.92	0.612	0.79	0.33-1.90	0.594
24–59 months				0.66	0.26-1.68	0.382	0.66	0.26-1.68	0.379
**Health conditions**									
Other developmental disabilities				Ref.			Ref.		
Cleft lip/palate				0.34	0.09-1.28	0.111	0.36	0.10-1.32	0.123
Cerebral palsy				0.74	0.21-2.65	0.649	0.76	0.21-2.71	0.669
**Reported infection**									
No							Ref.		
Yes							1.26	0.72-2.22	0.421
**Number of health conditions**									
One							Ref.		
Two or more							1.14	0.52-2.54	0.739
**Post-estimation and goodness-of-fit tests**									
Hosmer–Lemeshow test				H-L $\chi^2_{(7)}$=2.10; *p*-value: 0.954			H-L $\chi^2_{(7)}$=2.49; *p*-value: 0.928		
Area under ROC curve				0.690			0.692		
AIC				348.71			352.00		
BIC				378.83			389.65		
Likelihood-ratio test (Model 3 vs. 2)				LR $\chi^2_{(2)}$=0.71; *p*-value: 0.701					

AIC: Akaike's information criterion; AOR: Adjust odds ratio; BIC: Bayesian information criterion; H-L: Hosmer–Lemeshow; LR: Likelihood-ratio; OR: odds ratio; Ref: reference group; ROC: Receiver Operating Characteristic; SE: Standard error; WL/HZ: Weight-for-length/height-for-age *z*-score
P-values shown in bold are statistically significant (< 0.05).
