# Supplementary material for: Undernutrition and Feeding Difficulties Among Children with Disabilities in Uganda: A Cross-Sectional Study
Source: Nutrients. 2026 Jan 8;18(2):200. doi: 10.3390/nu18020200 (PMC12844944; doi:10.3390/nu18020200)
Supplement: Supplementary file 1 [file nutrients-18-00200-s001.zip › Nutrients_Supplementary Materials_TableS7.pdf]

Supplementary Materials

Table S7. Logistic regression models with post-estimation and goodness-of-fit tests for the association of risk for feeding difficulties with underweight in infants < 12 months old with cleft lip and/or palate (n=173)

	**Underweight (WAZ)**											
	**Model 1 (unadjusted)**			**Model 2 (demographics)**			**Model 3 (demographics + feeding practices)**			**Model 4 (demographics + feeding practices + health)**		
**Variables**	**OR**	**95% CI**	***p*-Value**	**AOR**	**95% CI**	***p*-Value**	**AOR**	**95% CI**	***p*-Value**	**AOR**	**95% CI**	***p*-Value**
**Risk for feeding difficulties**												
No	Ref.			Ref.			Ref.			Ref.		
Yes	1.39	0.75-2.60	0.297	2.40	1.11-5.18	**0.026**	2.78	1.16-6.66	**0.022**	2.70	1.13-6.48	**0.026**
**Sex**												
Female				Ref.			Ref.			Ref.		
Male				1.43	0.76-2.71	0.266	1.25	0.63-2.30	0.514	1.31	0.67-2.59	0.429
**Age groups**												
< 6 mo				Ref.			Ref.			Ref.		
6-11 months				0.36	0.16-0.85	**0.020**	0.27	0.13-0.78	**0.006**	0.28	0.11-0.72	**0.008**
**Breastfed**												
No							Ref.			Ref.		
Yes							0.46	0.21-0.98	0.055	0.46	0.21-1.03	0.059
**Bottle-fed**												
No							Ref.			Ref.		
Yes							2.08	1.03-4.99	0.085	2.01	0.86-4.67	0.104
**Feeding practices**												
Less than ideal							Ref.			Ref.		
Ideal							0.61	0.29-1.27	0.185	0.60	0.29-1.27	0.184
**Number of health conditions**												
One										Ref.		
Two or more										2.09	0.65-6.69	0.213
**Post-estimation and goodness-of-fit tests**												
Hosmer–Lemeshow test				H-L $\chi^2_{(4)}$=1.53; *p*-value: 0.821			H-L $\chi^2_{(8)}$=9.43; *p*-value: 0.308			H-L $\chi^2_{(8)}$=6.61; *p*-value: 0.579		
Area under ROC curve				0.62			0.71			0.72		
AIC				231.92			222.45			222.87		
BIC				244.54			244.52			248.09		
LR test (Model 3 vs. 2)				LR $\chi^2_{(3)}$=15.47; *p*-value**: 0.002**								
LR test (Model 4 vs. 3)							LR $\chi^2_{(1)}$=1.58; *p*-value: 0.208					

AIC: Akaike's information criterion; AOR: Adjust odds ratio; BIC: Bayesian information criterion; H-L: Hosmer–Lemeshow; LR: Likelihood-ratio; OR: odds ratio; Ref: reference group; ROC: Receiver Operating Characteristic; WAZ: weight-for-age *z*-score

P-values shown in bold are statistically significant (< 0.05).
