# Supplementary material for: Undernutrition and Feeding Difficulties Among Children with Disabilities in Uganda: A Cross-Sectional Study
Source: Nutrients. 2026 Jan 8;18(2):200. doi: 10.3390/nu18020200 (PMC12844944; doi:10.3390/nu18020200)
Supplement: Supplementary file 1 [file nutrients-18-00200-s001.zip › Nutrients_Supplementary Materials_TableS8.pdf]

Supplementary Materials

Table S8. Logistic regression models with post-estimation and goodness-of-fit tests for the association of risk for feeding difficulties with stunting in infants < 12 months old with cleft lip and/or palate (n=166)

	**Stunting (L/HAZ)**											
	**Model 1 (unadjusted)**			**Model 2 (demographics)**			**Model 3 (demographics + feeding practices)**			**Model 4 (demographics + feeding practices + health)**		
**Variables**	**OR**	**95% CI**	***p*-Value**	**AOR**	**95% CI**	***p*-Value**	**AOR**	**95% CI**	***p*-Value**	**AOR**	**95% CI**	***p*-Value**
**Risk for feeding difficulties**												
No	Ref			Ref			Ref			Ref		
Yes	1.63	0.85-3.14	0.143	2.49	1.01-4.86	**0.027**	3.24	1.31-7.99	**0.011**	3.27	1.30-8.21	**0.012**
**Sex**												
Female				Ref			Ref			Ref		
Male				1.01	0.52-1.96	0.978	0.86	0.43-1.74	0.685	0.98	0.48-2.01	0.950
**Age**												
< 6 mo				Ref			Ref			Ref		
6-11 months				0.44	0.18-1.06	0.068	0.45	0.17-1.18	0.103	0.46	0.17-1.22	0.121
**Breastfed**												
No							Ref			Ref		
Yes							0.79	0.35-1.79	0.577	0.80	0.35-1.84	0.598
**Bottle-fed**												
No							Ref			Ref		
Yes							3.35	1.37-8.18	**0.008**	3.09	1.24-7.70	**0.015**
**Feeding practices**												
Less than ideal							Ref			Ref		
Ideal							0.65	0.30-1.40	0.276	0.63	0.28-1.40	0.259
**Number of health conditions**												
One										Ref		
Two or more										5.10	1.42-18.34	**0.013**
**Post-estimation and goodness-of-fit tests**												
Hosmer–Lemeshow test				H-L $\chi^2_{(4)}$=2.87; *p*-value: 0.579			H-L $\chi^2_{(8)}$=3.75; *p*-value: 0.879			H-L $\chi^2_{(8)}$=1.56; *p*-value: 0.992		
Area under ROC curve				0.57			0.70			0.73		
AIC				215.87			208.05			203.10		
BIC				228.32			229.83			228.00		
LR test (Model 2 vs. 3)				LR $\chi^2_{(2)}$=13.82; *p*-value: **0.003**								
LR test (Model 3 vs. 4)							LR $\chi^2_{(1)}$=6.95; *p*-value: **0.008**					

AIC: Akaike's information criterion; AOR: Adjust odds ratio; BIC: Bayesian information criterion; H-L: Hosmer–Lemeshow; L/HAZ: Length/height-for-age *z*-score; LR: Likelihood-ratio; OR: odds ratio; Ref: reference group; ROC: Receiver Operating Characteristic

P-values shown in bold are statistically significant (< 0.05).
