# Supplementary material for: Undernutrition and Feeding Difficulties Among Children with Disabilities in Uganda: A Cross-Sectional Study
Source: Nutrients. 2026 Jan 8;18(2):200. doi: 10.3390/nu18020200 (PMC12844944; doi:10.3390/nu18020200)
Supplement: Supplementary file 1 [file nutrients-18-00200-s001.zip › Nutrients_Supplementary Materials_TableS9.pdf]

Supplementary Materials

Table S9. Comparison of undernutrition indicators and risk for feeding difficulties across three levels of challenges[1] (n=16)

	**Sufficient support in all domains n=5**	**Challenges in some of the domains n=6**	**Challenges in all domains n=5**	***p*-Value**
	**n/N (%)**	**n/N (%)**	**n/N (%)**	
Underweight (WAZ)	1/5 (20.0)	2/6 (33.3)	2/5 (40.0)	0.785
Stunting (L/HAZ)	1/5 (20.0)	1/5 (20.0)	1/5 (20.0)	1.000
Wasting (WL/HZ))	0/5 (0.0)	1/5 (20.0)	3/5 (60.0)	0.092
Risk for feeding difficulties	1/5 (20.0)	3/6 (50.0)	4/5 (80.0)	0.165

L/HAZ: Length/Height-for-age *z*-score; MUAC: Mid-upper arm circumference; WAZ: Weight-for-age *z*-score; WL/HZ: Weight-for-length/height *z*-score. Statistical analyses using Pearson's chi-squared. *P*-values shown in bold are statistically significant (< 0.05).

[1]Families were scored on three domains – access to services, access to food, and feeding difficulties – using a scale of 0 = insufficient access or current challenges or 1 = sufficient access or no current challenges. Scores across the three domains were summed to generate an overall challenge score ranging from 0 (challenges in all domains) to 3 (sufficient support in all domains).
